# Selective laser trabeculoplasty following failed combined phacoemulsification cataract extraction and excimer laser trabeculotomy can control intraocular pressure for a limited time

**DOI:** 10.1007/s10792-021-02039-x

**Published:** 2022-02-03

**Authors:** Iwona Krzyzanowska, Johanna Ziegler, Frances Meier-Gibbons, Marc Töteberg-Harms

**Affiliations:** 1 Medical Faculty, University of Zurich, Pestalozzistrasse 3, 8091 Zurich, Switzerland; 2 Eye Center Rapperswil, Merkurstrasse 50, 8640 Rapperswil, Switzerland; 3 Department of Ophthalmology, University Hospital Zurich, Frauenklinikstrasse 24, 8091 Zurich, Switzerland; 4 Augusta University, Medical College of Georgia, 1120 15th Street, Augusta, GA USA

**Keywords:** Glaucoma, Cataract, Minimally invasive glaucoma surgery, Excimer laser trabeculotomy, Selective laser trabeculoplasty

## Abstract

**Purpose:**

To assess the efficacy of selective laser trabeculoplasty (SLT) following failed phacoemulsification cataract extraction combined with excimer laser trabeculotomy (phaco-ELT).

**Methods:**

Retrospectively, the medical records of patients with primary or secondary open-angle glaucoma or ocular hypertension who underwent SLT between January 2001 and February 2015 by one surgeon at a single center after a failed phaco-ELT were evaluated. Exclusion criteria were: angle-closure glaucoma, optic nerve atrophy due to disease other than glaucoma, and additional glaucoma procedures between phaco-ELT and SLT. The main outcome measures were time to failure and Kaplan–Meier survival. Complete success was defined as a reduction of intraocular pressure (IOP) of  > 3 mmHg and  > 20% compared to baseline, and the number of AGM ≤ baseline.

**Results:**

A total of 23 eyes of 21 subjects were included. Baseline IOP was 19.7 (range, 19.1–22.7) mmHg, and the number of AGM at baseline was 2.5 (range, 1.9–2.9). Median time to failure after SLT was 7.2 (range, 6.6–7.8) months. The number of antiglaucoma medications did not change during that time.

**Conclusions:**

In eyes in which the IOP is no longer controlled following phaco-ELT, SLT could be an option to slow disease progression or prolong time until incisional filtration surgery. However, time to failure after SLT is limited. Thus, close follow-up visits are necessary in order to not delay an incisional surgery.

## Introduction

Glaucoma is one of the leading causes of irreversible blindness worldwide [1]. Glaucoma is a chronic, progressive neuro-degenerative disease characterized by progressive loss of retinal nerve fiber tissue leading to deterioration of the visual field and, ultimately, to blindness [2]. If treated early, disease progression and, thus, vision loss can be slowed down or even prevented. Currently, the only evidence-based treatment is the lowering of intraocular pressure (IOP) [3, 4]. Usually, topical IOP lowering medications (AGM) are the first-line therapy. However, during the course of the treatment, multiple drugs are required to reach a target pressure, which sufficiently slows down progression.

When AGM reach their therapeutic limits or are not tolerated by the patient, a surgical procedure is necessary to further lower IOP. Trabeculectomy has remained the gold standard in glaucoma surgery for more than five decades [5–7]. As the prevalence of glaucoma rises with age [1] and, thus, coexisting cataract is commonly observed, trabeculectomy combined with phacoemulsification cataract extraction has become an option in the armamentarium of surgical glaucoma management [8–10]. However, trabeculectomy combined with cataract surgery poses significant risks for the patient, such as hypotony and infections, i.e., blebitis with or without endophthalmitis [11, 12]. Throughout previous decades, various surgical procedures with a moderate efficacy and a more favorable safety profile compared to trabeculectomy have evolved and proven to be effective in lowering IOP, i.e., micro-invasive glaucoma surgery (MIGS) procedures. Hence, MIGS procedures are performed more often at earlier disease stages [13–15]. MIGS procedures can be performed as stand-alone or combined with cataract surgery [13–15].

Excimer laser trabeculotomy (ELT) is a MIGS procedure, which enhances physiological aqueous humor outflow by non-thermally creating micro-perforations (laser channels) with an excimer laser from the anterior chamber through the trabecular meshwork (TM) and inner wall of Schlemm’s canal into Schlemm’s canal (conventional/trabecular outflow) [11, 16–21]. ELT is usually combined with phacoemulsification cataract surgery (phaco-ELT) [11, 16–21]. ELT does not require an implant unlike other MIGS procedures.

Selective laser trabeculoplasty (SLT) is a non-invasive office procedure, which targets the trabecular meshwork as well and involves the delivery of electromagnetic energy to the TM to enhance aqueous drainage and, thus, lower intraocular pressure [22]. SLT is performed with a Q-switched, frequency-doubled neodymium-yttrium–aluminum-garnet laser (Nd:YAG; wavelength, 532 nm). Since the introduction of SLT by Mark Latina in the mid-1990s, the exact mechanism of action remains unknown [23, 24]. Besides the broad availability and the favorable risk profile of SLT, a major advantage of this technique is its repeatability [25]. A post hoc analysis of the data from the Laser in Glaucoma and Ocular Hypertension (LiGHT) trial demonstrates that repeated SLT can maintain IOP at or below target IOP in medication-naive open-angle glaucoma and ocular hypertension requiring retreatment with at least an equivalent duration of effect to the initial laser intervention [25]. SLT is increasingly performed in glaucoma management in recent years. Thus, it is of utter importance to question its efficacy as a subsequent intervention in cases where other glaucoma surgeries, e.g., ELT, have failed.

The aim of this study was to investigate whether SLT could still be an effective IOP-lowering treatment option after failed phaco-ELT before considering other more invasive procedures, such as incisional filtration surgery, e.g., trabeculectomy.

## Material and methods

This study was approved by the institutional review board (cantonal ethics committee of the canton of Zurich, Zurich, Switzerland, KEK No. 2015–00,193). All study-related analyses were conducted in adherence to the Helsinki Declaration and national laws. A waiver was granted for informed consent. Retrospectively, medical records of all patients who underwent SLT after a failed phaco-ELT between January 2001 and February 2015 performed by one surgeon at the Department of Ophthalmology at the University Hospital Zurich, Switzerland, were evaluated.

### Inclusion and exclusion criteria

Inclusion criteria were the age of > / = 18 years with no upper limit, a diagnosis of primary or secondary open-angle glaucoma or ocular hypertension, and a history of combined phaco-ELT surgery. Definition of failure for phaco-ELT was: an individual target pressure was not reached at two consecutive visits or the glaucoma was further progressing besides an IOP within the target range. All eyes must have undergone SLT after phaco-ELT between January 2001 and February 2015. Exclusion criteria were angle-closure glaucoma, optic nerve damage not caused by glaucoma, history of any other glaucoma procedure between phaco-ELT and SLT, SLT prior to phaco-ELT, and refused written informed consent.

### Combined phacoemulsification and excimer laser trabeculotomy technique

Phaco-ELT is a combined procedure involving cataract surgery and ELT. ELT was performed directly after cataract surgery (standard clear cornea-phacoemulsification and intracapsular lens implantation, Alcon MA50BM, Alcon Inc., Hünenberg, Switzerland). First, medical miosis was induced using acetylcholine, and the anterior chamber was deepened by inserting a high viscoelastic agent (Natriumhyaluronat, Healon). The laser fiber was inserted through a paracentesis and advanced across the anterior chamber under gonioscopic guidance. Ten laser channels with a diameter of approximately 0.5 mm were spaced over 90 degrees into the trabecular meshwork using an excimer laser (Excimer Laser, AIDA, TUI-Laser, Munich, Germany; pulse energy 1.2 mJ, pulse duration 60 ns, wavelength 308 nm). The successful application causes trabecular meshwork ablation. A formation of bubbles along with commonly observed blood reflux, which occurs when IOP is below episcleral venous pressure during phaco, proves the successful penetration into Schlemm’s canal and usually ceases after removal of the viscoelastic agent and spontaneous toning of the bulbus to 15 mmHg or above. The technique has been described previously [12, 19, 20, 26, 27].

### Selective laser trabeculoplasty technique

SLT was performed with a frequency-doubled Q-switched Nd:YAG laser (Ellex Tango Laser, Ellex Medical Laser, Adelaide, Australia; wavelength 532 nm, pulse duration 3 ns, spot size 400 μm). Using an Ocular Latina SLT Gonio Laser lens (Ocular Inc., Bellevue, Washington, USA), the laser was focused onto the pigmented trabecular meshwork and electromagnetic energy to the trabecular meshwork was delivered. The energy was titrated starting at 0.8 mJ in 0.1 mJ increments until “champagne bubbles” were visible. Before the procedure, a topical alpha-agonist (brimonidine 0.2%) and topical anesthesia (tetracaine 1.0%) were administered to the treated eye. Then, approximately 100 non-overlapping laser spots were delivered over 360°. The technique has been previously described [28].

### Baseline and follow-up

Baseline and follow-up patient data were retrospectively collected from medical records, including best-corrected visual acuity (BCVA), IOP, and AGM. Study data were collected for each patient once before SLT (baseline) and then at 1 day, 1 week, 1, 2, 3, 6, 12, 18, and 24 months after SLT, and subsequently every year until 60 months after SLT. At each study visit, BCVA, IOP, and the number of AGM were collected for each patient.

### Definitions of failure and success

Complete success was defined as a reduction of IOP of > 3 mmHg and > 20% compared to baseline, and the number of AGM ≤ baseline [28]. Qualified success 1 was defined as a reduction of IOP > 3 mmHg and > 20% compared to baseline, and qualified success 2 was defined as a reduction of IOP > 20% compared to baseline. Success was testified when the criteria were met either for complete or qualified success 1 or 2. Failure was the opposite of success. Definition of failure also included any additional glaucoma surgery during follow-up or loss of light perception vision. Date of failure was the first date out of 2 consecutive follow-up visits on which the definitions of success have not been met starting with 1 month after SLT. In case of alternating success and failure, the last follow-up visit determined success or failure. For demographic data, analysis data collected at 1 day and 1 week after SLT were also included.

### Statistical methods

All data were coded in Excel 2013 (Microsoft Office, Redmond, Washington, USA) and analyzed with SPSS version 22.0 (IBM Corporation, Armonk, New York, USA). Descriptive statistics such as median, interquartile range, mean and standard deviation (95% confidence interval) for continuous variables and absolute and relative frequencies for categorical variables were computed. Paired samples Wilcoxon test was used to determine significant differences in BCVA, IOP, and AGM, for intra-patient comparison at each follow-up visit compared to baseline. Kaplan–Meier survival analysis was used to assess time to failure described by median survival and tested by Logrank and Breslow-Gehan test. In addition, the multiple Cox regression was used to investigate potential confounders with regard to time to failure (gender, age). A *p*-value less than 0.05 was considered to be statistically significant. All visual acuities were converted to logarithm of the minimal angle of resolution (logMAR) visual acuity for statistical analysis.

## Results

**Fig. 1:**
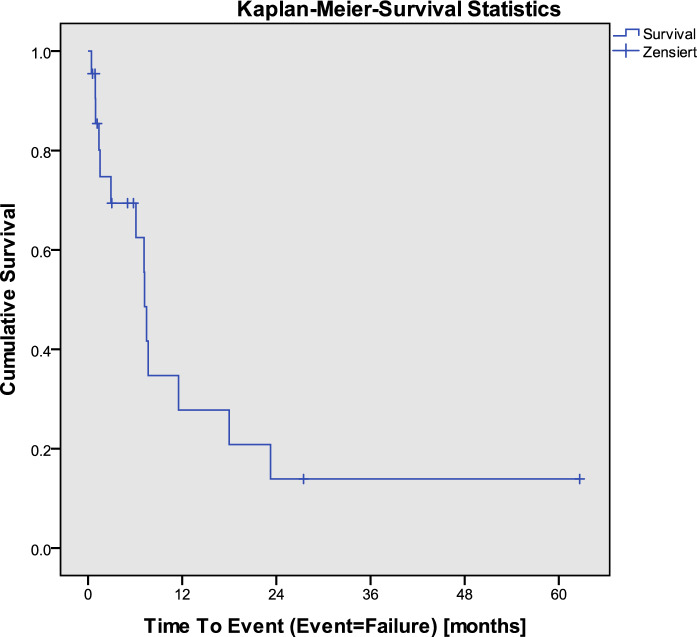
Kaplan-Meier-Survival plot for complete success

Phaco-ELT was performed on 283 eyes of 223 patients between January 2001 and February 2015 by one surgeon at the Department of Ophthalmology at the University Hospital Zurich, Switzerland. Among these, 255 eyes did not receive SLT after phaco-ELT and, thus, were excluded. Four eyes were not included because of patients’ refusal to participate in research. One patient was excluded due to having a diagnosis other than the inclusion diagnoses, i.e., angle-closure glaucoma. One patient was excluded due to missing follow-up data. The data were still included for the analysis of demographic and baseline data, but not for Kaplan–Meier survival analysis. Finally, a total of 23 eyes of 21 patients were included for statistical analyses (15 eyes [65.2%] of female patients and 8 eyes [34.8%] of male patients). Among these, nine eyes (39.1%) had a diagnosis of pseudoexfoliation glaucoma, and one eye (4.3%) had juvenile glaucoma. Mean age was at the time of phaco-ELT 75.0 (range, 71.3–78.7) years (Table 1). Median time to failure of phaco-ELT was 1.0 (range, 0.5–1.5) years. No influence on time to failure of phaco-ELT was found by gender (women, median time to failure 1.0 [range, 0.5–1.5], men, median time to failure 0.7 [range, 0.4–1.1], *p*-value 0.2), or age (Hazard Ratio 1.0, *p*-value 0.4). Mean baseline BCVA was 0.1 (range, 0.02–0.18), mean baseline AGM 2.45 (range, 1.97–2.94), and mean baseline IOP 19.74 (range, 19.05–22.74) (Table 1). Each eye included was treated with SLT after failed phaco-ELT. Mean number of spots were 100.2 (range, 61.6–138.8) at a mean total energy of 84.2 mJ (range, 48.4–119.9). Among these, two patients required additional glaucoma surgery after SLT, one received transscleral cyclophotocoagulation and one received a trabeculectomy with mitomycin C during the follow-up, which were counted as treatment failure. There was no occurrence of loss of light perception vision during the entire follow-up. Changes in IOP and AGM are shown in Table 2. Median survival time after SLT until failure was 7.2 (range, 6.6–7.8) months (Fig. 1). Survival data is shown in Table 3. Females had a significantly longer time to failure compared to males. No influence of age on survival could be shown. At 6 months 69.4% had complete success.

**Table 1:** Demographic data of patients who received selective laser trabeculoplasty following failed phaco-ELT

Characteristic	Value
Age at phaco-ELT (years)	75 (71.3–78.7)*
*Gender*	
Female	15 eyes (65.2%)
Male	8 eyes (34.8%)
*Glaucoma diagnosis*	
Pseudoexfoliative glaucoma	9 eyes (39.1%)
Juvenile glaucoma	1 eye (4.3%)
Open-angle glaucoma or ocular hypertension	13 eyes (56.6%)
BCVA (logMAR)	0.1 (0.02–0.18)*
AGM	2.45 (1.97–2.94)**
IOP	19.74 (19.05–22.74)**

*Mean (range) AGM = antiglaucoma medications; BCVA = best-corrected visual acuity; IOP = intraocular pressure; LogMAR = logarithm of the minimal angle of resolution; Phaco-ELT = phacoemulsification cataract extraction combined with excimer laser trabeculotomy

**Table 2:** Change in IOP, AGM, and BCVA (Δ IOP, Δ AGM, and Δ BCVA) at 1 day, 1 week, 1, 2, 3, 6, 12, 18, 24, 36, and 48 months after SLT compared to baseline using paired samples Wilcoxon test (Wilcoxon signed-rank test)

Time of visit	Δ IOP [mmHg]	*p*-value	Δ AGM [n]	*p*-value	Δ BCVA [logMAR]	*p*-value
1d	− 1.0	0.326	− 1.3	0.194	− 0.0	1.000
1w	− 2.2	0.027	− 1.0	0.317	− 1.6	0.109
1 m	− 2.5	0.012	− 4.5	0.655	− 1.5	0.144
2 m	− 1.1	0.279	− 0.0	1.000	− 1.1	0.285
3 m	− 1.9	0.056	− 0.0	1.000	− 1.3	0.197
6 m	− 1.9	0.580	− 0.8	0.414	− 1.0	0.317
12 m	− 0.5	0.646	− 0.4	0.705	− 1.8	0.066
18 m	− 0.9	0.345	− 0.0	1.000	− 0.4	0.655
24 m	− 0.0	1.000	− 1.0	0.317	− 0.5	0.655
36 m	− 1.3	0.180	− 0.0	1.000	− 1.0	0.317
48 m	− 1.6	0.109	− 1.4	0.157	− 1.0	0.317

(AGM = antiglaucoma medications; BCVA = best-corrected visual acuity; d = day; IOP = intraocular pressure; LogMAR = logarithm of the minimal angle of resolution; m = month/months; SLT = selective laser trabeculoplasty; w = week)

**Table 3:** Success rates of SLT computed by Kaplan–Meier survival analysis

Time of visit	Success rate of SLT
1 month	85.4%
2 months	74.7%
3 months	69.4%
6 months	69.4%
12 months	27.8%
18 months	20.8%
24 months	13.9%
36 months	13.9%
48 months	13.9%
60 months	13.9%

The success rate is defined as survival time after SLT until failure of SLT

SLT = selective laser trabeculoplasty

## Discussion

Median survival time of SLT after failed phaco-ELT in patients with primary or secondary open-angle glaucoma or ocular hypertension, and cataract was 7.2 (range, 6.6–7.8) months. Thus, SLT could be an additional effective treatment option among the growing range of glaucoma interventions that physicians could offer to patients after failed phaco-ELT. However, the duration of the IOP lowering effect of SLT after failed phaco-ELT was limited. Thus, a subsequent incisional IOP lowering surgery can only be delayed by SLT. Given that our study could show limited effectiveness of SLT after failed phaco-ELT, we have to acknowledge a few limitations, including the retrospective study design and low number of included eyes. Given the limitations by the retrospective study design, no sub-analyses of success based on glaucoma severity, or visual field data was possible. In addition, it would have been valuable to see whether the delay in subsequent incisional surgeries by the limited time of success after SLT would have positively influenced the quality of life of the patients. However, no data on the quality of life was available in medical records.

Previous studies already proved the effectiveness of SLT as a primary procedure as well as after various failed glaucoma procedures. Beltran-Agullo et al. reported that primary SLT significantly lowers IOP in patients with primary open-angle glaucoma and ocular hypertension from 24.0 ± 3.0 to 18.9 ± 2.7 mm Hg (*P* < 0.001) 3 months after laser treatment [22]. Birt investigated the one-year effect of SLT performed after failed 360 degrees of ALT in comparison to primary ALT or SLT in patients with open-angle glaucoma (primary, pigmentary, or pseudoexfoliation glaucoma) [29]. Birt showed a significant IOP reduction one year after SLT after prior ALT, in an extent similar to that of ALT. Russo et al. also proved SLT to be as effective in IOP lowering as ALT in patients with uncontrolled open-angle glaucoma on maximally tolerated medication therapy at 12 months with no prior intervention, and in the case of retreatment, SLT appeared to be more effective than ALT in IOP lowering [30]. We hypothesize that the effectiveness of SLT in this study is limited compared to primary SLT as the prior performed ELT procedure has altered the TM and, thus, the effect of subsequent SLT after failed phaco-ELT is limited due to the lesser amount of treatmet-naïve TM.

A similar study to the present study by Töteberg-Harms and Rhee investigated the effect of SLT following failed phacoemulsification cataract surgery combined with ab interno trabeculectomy (phaco-AIT) using the trabectome in patients with primary open-angle glaucoma or pseudoexfoliation glaucoma [28]. In this study, SLT had a very low success rate with only a median time to failure of 3.6 months. Contrarily, the time to failure of SLT after phaco-ELT was approximately twice as long compared to after phaco-AIT. A potential explanation is that the trabectome removes a huge amount of TM of approximately 120° while ELT removes only a little amount of TM within 90° (10 laser channels with a diameter of approximately 400 µm each). Thus, there is more remaining TM to perform SLT after phaco-ELT compared to after phaco-AIT.

Based on these findings, SLT will not effectively lower IOP for a long time after failed phaco-ELT. Thus, fistulating surgery should be offered to patients when a phaco-ELT fails as it will lower IOP more significantly and for a longer amount of time. However, SLT can still be considered to lower IOP for a few months in cases where patients refuse trabeculectomy, especially due to the broad availability of SLT and its relatively favorable safety profile. Patients must be advised, that fistulating surgery cannot be postponed for a long time by SLT after failed phaco-ELT.

## Declarations

### Data availability

The datasets generated and analyzed during the current study are available from the corresponding author on reasonable request.
